# Differential gene expression in Lin^-^/VEGF-R2^+^ bone marrow-derived endothelial progenitor cells isolated from diabetic mice

**DOI:** 10.1186/1475-2840-13-42

**Published:** 2014-02-12

**Authors:** Daniel Barthelmes, Ling Zhu, Weiyong Shen, Mark C Gillies, Mohammad R Irhimeh

**Affiliations:** 1 Save Sight Institute, Level 1, South Block Sydney Hospital and Sydney Eye Hospital, Central Clinical School, The University of Sydney, 8 Macquarie Street, Sydney, NSW 2000, Australia

**Keywords:** Diabetes, Endothelial progenitor cells, Diabetic vasculopathies, Molecular pathology, SDF-1, Lin^-^/VEGF-R2^+^ EPCs, Cardiovascular pathology, Retinopathy

## Abstract

**Background:**

Diabetes is known to impair the number and function of endothelial progenitor cells in the circulation, causing structural and functional alterations in the micro- and macro-vasculature. The aim of this study was to identify early diabetes-related changes in the expression of genes that have been reported to be closely involved in endothelial progenitor cell migration and function.

**Methods:**

Based on review of current literature, this study examined the expression level of 35 genes that are known to be involved in endothelial progenitor cell migration and function in magnetically sorted Lin^-^/VEGF-R2^+^ endothelial progenitor cells obtained from the bone marrow of Akita mice in the early stages of diabetes (18 weeks) using RT-PCR and Western blotting. We used the Shapiro-Wilk and D’Agostino & Pearson Omnibus tests to assess normality. Differences between groups were evaluated by Student’s t-test for normally distributed data (including Welch correction in cases of unequal variances) or Mann–Whitney test for not normally distributed data.

**Results:**

We observed a significant increase in the number of Lin^-^/VEGF-R2^+^ endothelial progenitor cells within the bone marrow in diabetic mice compared with non-diabetic mice. Two genes, SDF-1 and SELE, were significantly differentially expressed in diabetic Lin^-^/VEGF-R2^+^ endothelial progenitor cells and six other genes, CAV1, eNOS, CLDN5, NANOG, OCLN and BDNF, showed very low levels of expression in diabetic Lin^-^/VEGF-R2^+^ progenitor cells.

**Conclusion:**

Low SDF-1 expression may contribute to the dysfunctional mobilization of bone marrow Lin^-^/VEGF-R2^+^ endothelial progenitor cells, which may contribute to microvascular injury in early diabetes.

## Background

The inner lining of blood vessels, the endothelium, is made up of a single layer of endothelial cells (ECs) [1], which acts as a barrier between the blood and the surrounding tissue. It prevents inflammatory cell infiltration, modulates vascular tone and controls smooth muscle cell proliferation [2-4]. Damage to the endothelium can be repaired by proliferation and migration of nearby mature ECs [5,6], which have limited regenerative capacity [7,8].

Endothelial progenitor cells (EPCs) originating from the bone marrow (BM) can migrate to the peripheral blood (PB) [9,10] and repair injured endothelium [11,12]. These EPCs play an important role in regenerating the endothelium through migration, proliferation, differentiation and by secreting pro-angiogenic cytokines [13]. EPCs express a range of cell surface markers, among them stem cell markers (CD34, CD133) and endothelial markers (CD146, vWF, VEGF-R2) [14,15]. As no single or unique marker for EPCs has been identified, researchers use a range of markers and phenotypical properties to define them [16,17]. Regardless of this lack of accuracy, the term "EPC" is used and it is acknowledged that "EPC" refers to a heterogeneous population of cells rather than a single population [17-19].

Diabetes mellitus, characterized by chronic hyperglycemia [20], is a metabolic condition that strongly affects EPCs. Diabetic patients have reduced numbers of EPCs [21,22] in the PB and the function of such EPCs isolated from PB with respect to proliferation, tube formation and adhesion is impaired [23,24]. Most importantly, EPCs from diabetic individual are less competent in repairing vascular injuries [23,25,26]. Not only impaired function but also reduced mobilization from the BM to the PB has been documented [22,24] in diabetic patients. Both the decreased number of EPCs and their impaired function have been proposed to be involved in the pathogenesis of vascular complications in diabetes [21,23,27].

Since most studies to elucidate molecular mechanisms of EPC impairment in diabetes have been conducted in EPCs isolated from the PB in humans with a long history of diabetes, little is known about the changes occurring in EPCs located within the BM in the early stages of diabetes. A subset of BM derived EPCs, which are phenotypically characterized as Lin^-^/VEGF-R2^+^ cells were recently described [28]. These Lin^-^/VEGF-R2^+^ endothelial progenitor cells (abbreviated as Lin^-^/VEGF-R2^+^ EPCs in this study) have typical properties of EPCs such as formation of cobblestone-shaped colonies, Dil-acLDL uptake, lectin binding, expression of typical EPC markers such as VEGF-R2 and CD34, lack of expression of CD31, CD45, CD14 and CD115 and incorporation into damaged blood vessels *in vivo*[28] .

We have recently shown [28] that BM derived Lin^-^/VEGF-R2^+^ cells isolated from diabetic mice showed neither functional differences nor reduced proliferative capacity compared with such cells isolated from non-diabetic mice. However, we found a distinct defect in mobilization of Lin^-^/VEGF-R2^+^ cells from BM to the PB in diabetic mice. To explore the molecular mechanisms underlying this defect in mobilization of BM Lin^-^/VEGF-R2^+^ EPCs in spontaneously diabetic mice, the current study aimed to evaluate differential gene expression of 35 genes that were reported to be closely involved in Lin^-^/VEGF-R2^+^ EPC mobilization and function.

## Methods

### Animals

All animal studies were conducted in accordance with the New South Wales Animals Act (1985). Approval was issued by The University of Sydney Animal Ethics Committee (Approval number: K17/9-2007/3/4664). All efforts were made to minimize animals suffering. The animals, Ins2^Akita^ mice (Akita mice), were obtained from The Jackson Laboratory (Bar Harbor, ME, USA). The Akita mouse carries a dominant point mutation in the Insulin 2 gene on chromosome 7 resulting in the development of diabetes at approximately 4 weeks after birth [29] with almost 100% penetrance [30]. As female mice develop diabetes more slowly and less stably compared with males [29], only male mice heterozygous for the Ins2^Akita^ allele (diabetic group) as well as male mice homozygous for the wild type Ins2 allele (non-diabetic mice) were used in this study. Presence of the Ins2^Akita^ allele or the wild type Ins2 gene was confirmed by RFLP analysis [30]. Once diabetes was established (blood glucose level > 13.3mmol/l [31]), mice were monitored weekly for changes in body weight and blood glucose levels for 18 weeks. The blood glucose level was measured using Accu-Chek Performa (Roche, Germany). No supplemental insulin was given. Only mice having blood glucose levels consistently ≥ 13.3 mmol/l were used in this study. Eight diabetic and 8 non-diabetic mice were used.

### BM collection

After euthanizing mice with CO_2_, the femorae and tibiae of both legs were immediately excised and the diaphyses flushed using a 25g needle and 8 ml of IMag™ buffer (BD, Cat no. 552362). The collected cells were placed on ice. After centrifugation (400 rcf, 5 min), the cell pellet was resuspended in 2 ml red cell lysing buffer (Sigma, Cat no. R7757). After 5 min incubation and centrifugation (400 rcf, 5 min), the cell pellet was resuspended in 10 ml of IMag™ buffer and washed twice. Cells were eventually filtered using a 40 μm nylon cell strainer (BD, Cat no. 352340), centrifuged (400 rcf, 5 min) and resuspended in 2 ml of IMag™ buffer. After cell counting (TC10, BioRad) and viability assessment using the trypan blue exclusion assay, cells were placed on ice for the isolation of Lin^-^/VEGF-R2^+^ cells.

### Immunomagnetic bead separation of BM Lin^-^/VEGF-R2^+^ endothelial progenitor cells

BM cells were incubated with NA/LE rat anti-mouse CD16/CD32 (Fc-block, 1 μg/10^6^ cells, BD, Cat no. 553140) for 15 min. After Fc-block, BM cells were incubated with a solution containing an APC mouse lineage antibody cocktail (BD, Cat no. 558074) and a FITC rat anti-mouse Flk-1/VEGF-R2 antibody (BD, Cat no. 560680). After 20 min incubation, cells were centrifuged (400 rcf, 5 min) and washed twice using cold IMag™ buffer. Cells were incubated with magnetic beads for 30 min at 4°C (APC magnetic particles-DM; BD, Cat no. 557932). The APC mouse lineage cocktail was used to separate Lin^+^ cells from the whole of bone marrow cells, i.e. hematopoietic lineage cells such as T lymphocyes, B lymphocytes, monocytes/macrophages, granulocytes, and erythrocytes cells containing surface antigens such as CD3e, CD11, CD45R/B220, Ly-76, Ly-6G and Ly-6C. The Lin^+^ depletion was conducted using a Dynal MPC-S magnetic separator (Invitrogen, Cat no. 12020D). The Lin^-^ fraction was further incubated with anti-FITC beads (Miltenyi Biotec, Cat no. 130-048-701) for 30 min at 4°C. The fraction of Lin^-^/VEGF-R2^+^ EPCs was obtained via a positive selection step using the magnetic separator. For RNA isolation, fractions of both Lin^+^ and the Lin^-^/VEGF-R2^+^ cells were collected, washed in PBS and centrifuged. Cell pellets were re-suspended in 100 μl of RNA later solution (Qiagen, Cat no. 76104) and snap-frozen in liquid nitrogen. Cells destined to undergo protein analysis were directly snap-frozen and stored at -80°C for further use.

### Group design

Four experimental groups were established: 1) Lin^+^ cells from non-diabetic animals, 2) Lin^+^ cells from diabetic animals, 3) Lin^-^/VEGF-R2^+^ cells from non-diabetic animals and 4) Lin^-^/VEGF-R2^+^ cells from diabetic animals. The Lin^+^ cells were used as an internal reference to identify differential gene expression occurring not exclusively in Lin^-^/VEGF-R2^+^ cells. This setup allowed us to distinguish differential gene expression which specifically occurred in diabetic BM derived Lin^-^/VEGF-R2^+^ EPCs from that which may also occur in other phenotypes of BM cells. Hence, only significant changes in gene expression observed in diabetic vs. non-diabetic Lin^-^/VEGF-R2^+^ EPCs that did not occur in the Lin^+^ population were considered in the final analysis.

### RNA isolation

RNA isolation was performed at room temperature using the RNeasy Mini Kit (Qiagen, Cat no. 74104) according to the manufacturer’s instructions. Isolated RNA was snap-frozen and stored at -80°C for further use. RNA concentration was measured using a Nanodrop 1000 (Nanodrop Products, DE, USA), integrity was assessed using the BioRad Experion automated electrophoresis system (BioRad, CA, USA) on a RNA StdSens Chip (BioRad Cat no. 700–7159).

### Reverse transcription

Reverse transcription was done using the iScript cDNA synthesis kit (BioRad, Cat no. 170–8890). In brief, 4 μl of 5× iScript reaction buffer, 1 μl iScript reverse transcriptase, 500 ng RNA and water up to a total reaction volume of 20 μl were mixed. The reverse transcription program was designed as follows: 25°C for 5 min, 42°C for 60 min, 85°C for 5 min followed by 4°C at a hold step. Reactions were performed in a PCR machine (HBPX220, Hybaid, UK). The finial 20 μl cDNA product was diluted into 160 μl total volume using MQ water.

### Real time PCR

Primer sequences for RT-PCR were obtained from http://pga.mgh.harvard.edu/primerbank and from http://primerdepot.nci.nih.gov/. *In silico* analyses were performed to identify the amplicon size and suitability of the primer pairs. An overview of genes tested and primers used is shown in Additional file 1: Table S1. All primers had a melting temperature of approximately 61°C and were tested before RT-PCR using gel electrophoresis to visualize amplicons. For testing primers, a total reaction volume of 10 μl comprised of 5 μl Super Mix (SsoFast EvaGreen Supermix, BioRad, Cat no. 172–5200), 1 μl of 4 μM forward and reverse primer mixture, 1 μl of cDNA and 3 μl water. PCR steps used were similar to the RT-PCR program used later: 95°C for 30 s, 40 cycles of 95°C for 5 s then 60°C for 20 s. This was followed by a melting curve step starting from 65°C to 95°C each step lasting 30s, ramp rate was 0.5°C/s. PCR products were analyzed in 2% agarose (in TBE buffer) gels to verify amplicon size.

RT-PCR was performed on a LightCycler 480 (Roche, Switzerland) using 384 well plates. Each group included seven individual samples, each individual sample was replicated once (technical replicate). The program was as follows: 95°C for 5 min, 40 cycles of 95°C for 10 s, 60°C for 20 s and 72°C for 20 s. Ramp rate was 4.8°C/s. Each well contained 5 μl Express Sybr Green (Invitrogen, Cat no. 10000162), 0.5 μl water, 0.5 μl of 4 μM forward and reverse primer mixture and 4 μl of the diluted sample cDNA. Mouse glyceraldehyde-3-phosphate dehydrogenase (mGAPDH) [32,33] and 18S ribosomal RNA (18srRNA) were used as reference genes. The two reference genes were chosen using "BESTKEEPER" software ( http://rest-2009.gene-quantification.info/), taking into account the information that there are no significant differences in mGAPDH and 18srRNA. Since progenitor cells from bone marrow were used, it was not clear whether one single chosen reference gene would be expressed. GAPDH content may be altered in animal models of diabetes, however, not all mouse strains are affected and in C57/BL6 mice mGAPDH has been successfully used as reference gene [32,33]. CT-values were computed using the 2nd order derivation method, CT values ≥ 35 were excluded from the analysis. Data analysis was performed using the RT^2^ profiler PCR array data analysis available on http://pcrdataanalysis.sabiosciences.com/pcr/arrayanalysis.php.

### Protein isolation and Western blot

For Western blot analysis, 8 samples from each group were used. The isolated cells were incubated and lysed for 30 min at 4°C in RIPA buffer (Sigma, Cat no. 127K6009) containing protease inhibitor (Complete mini; Roche, Cat no. 046931240010; 1 tablet per 10 ml RIPA buffer). Buffer volume was adjusted to a concentration of 5 × 10^4^ cells/μl RIPA buffer. The lysed cells were centrifuged at 12,000 rcf for 20 min at 4°C. The supernatant containing the protein was aliquoted (26 μl) and stored at -20°C for further use.

Gel-electrophoresis to separate proteins according to their size was done using 2,2-Bis(hydroxymethyl)-2, 2′, 2″-nitrilotriethanol (Bis-Tris) polyacrylamide gels with a gradient from 4 to 12% under denaturing conditions (Nupage, Invitrogen, Cat no. NP0335) using 2-(N-morpholino) ethanesulfonic acid sodium dodecyl sulfate (MES-SDS, Invitrogen, Cat No NP0002) as running buffer. Before loading the gel wells, 26 μl protein sample, 10 μl loading buffer (Invitrogen, Cat no. NP0007), 4 μl 500 mM DL-dithiothreitol (DTT; Sigma, Cat no. D9779-10G) were mixed and kept at 70°C for 10 min to denature the protein and after this kept on ice for 5 min. After gel-electrophoresis, gels were removed from the running chamber and placed on a 0.2 μm polyvinyl difluoride (PVDF) membrane (Invitrogen, Cat no. LC2002). The protein transfer was done using a wet transfer system (BioRad Mini Trans-Blot, Cat no. 170–3930). After the transfer, the PVDF membranes were washed for 5 min using water and then for 10 min using TBST: tris (hydroxymethyl) aminomethane (TRIS) buffer, 150 mM sodium chloride and 0.1% polyoxyethylene (20) sorbitan monolaurate (TWEEN 20). A blocking step followed using 5% bovine serum albumin (BSA, Sigma, Cat no. 9418) in TBST and incubating the PVDF membrane for 1 h at room temperature. After washing the membrane twice in TBST, the incubation with the primary antibody (SDF-1; 1:2000, Abcam, Cat no. ab25117) in TBST and 1% BSA followed over night at 4°C. The next day the primary antibody solution was removed and the PVDF membrane washed 3 times in TBST for 5 min. Exposure to the secondary antibody HRP-goat anti-rabbit IgG (H + L) conjugate (horseradish peroxidase coupled; Zymed, Cat no. 81–6120) followed for 2 h at room temperature. After washing 3× for 5 min with TBST, the PVDF membrane was washed twice with TBS and then incubated for 5 min with the chemoluminescent agent (Millipore, Cat no. WBKLS0500). Immediately after this, the chemoluminescent agent was removed and the PVDF membrane was analyzed using a digital imaging system (G:Box, Syngene, MD, USA). After recording, the PVDF membrane was stripped of the antibodies by incubating for 5 min at room temperature with a Western blot stipping buffer (Thermo Scientific, Cat no. 46430). After washing the membrane in TBST, the incubation with the next primary antibody followed (E-Selectin, 1:2000, Abcam, Cat no. ab18981) and the procedure of overnight incubation, secondary antibody incubation and imaging was repeated. To evaluate differences in protein levels, chemiluminosity readings of target proteins were divided by chemiluminosity readings of reference proteins from the same sample. Relative expression levels were compared between diabetic and non-diabetic samples.

### Statistics

Data are presented as mean ± standard deviation (SD) for normally distributed data and as mean [interquartile range] when non-normally distributed. Normality was assessed using the Shapiro-Wilk test and the D’Agostino and Pearson Omnibus normality tests. Differences in variances of normally distributed data were assessed using Levene’s test. Differences between two groups were either assessed using a student’s t-test (normally distributed data) including Welch correction in case of unequal variances or Mann–Whitney test (non-normally distributed data). Statistical significance was defined as p < 0.05.

## Results

### Animal body weight and blood glucose levels

**Figure 1:**
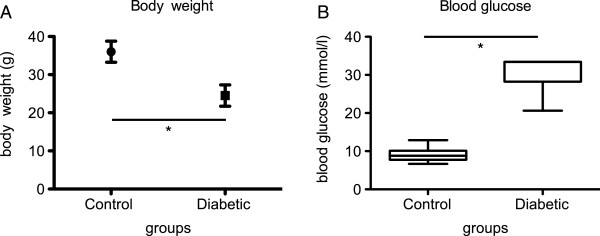
**Body weight and blood glucose levels.** Body weight **(A)** and blood glucose levels **(B)** in non-diabetic (control) and diabetic mice at 22 weeks of age (18 weeks of diabetes). Asterisk (*) denotes statistical significance (p < 0.05).

All heterozygous male Akita mice had blood glucose levels ≥13.3 mmol/l from 4 weeks of age. At the time of analysis, mice 22 weeks of age had a body weight of 36.0 ± 2.8 g and 24.5 ± 2.8 g for non-diabetic and diabetic mice respectively (p < 0.0001). Mean blood glucose at 22 weeks of age was 8.9 [7.8-10.2] mmol/l in non-diabetic and 33.4 [28.2-33.4] mmol/l in diabetic animals (p < 0.0001, Figure 1).

### Cell numbers in the bone marrow

Overall an absolute number of 3.33 [2.93-3.98] × 10^7^ nucleated cells were isolated from the BM of each mouse. On average, 5.4 ± 2.3% of the BM nucleated cells were Lin^-^/VEGF-R2^+^ cells and 82.3 ± 4.4% were Lin^+^. The remaining Lin^-^/VEGF-R2^-^ cells made up ~12%. While in non-diabetic mice 9.37 [8.61-10.75] × 10^5^ cells/g body weight were isolated, from diabetic mice 1.37 [1.10-1.69] × 10^6^ cells/g body weight could be obtained (p < 0.0001). Similarly, the number of Lin^-^/VEGF-R2^+^ EPCs in the BM was significantly greater in diabetic than non-diabetic animals after adjusting for body weight: 7.56 [6.20-8.37] × 10^4^ cells/g body weight vs. 4.24 [3.44-5.08] × 10^4^ cells/g body weight (p < 0.0001). There were 1.17 [0.94-1.39] × 10^6^ Lin^+^ cells/g body weight in diabetic animals and 7.58 [6.85-8.71] × 10^5^ Lin^+^ cells/g body weight in non-diabetic animals (p = 0.074).

### RNA quality and quantity

From the Lin^+^ fraction, 253.5 ± 117.3 ng/μl RNA could be isolated from diabetic animals and 234.0 ± 98.1 ng/μl RNA from non-diabetic animals (p = 0.64). The Lin^-^/VEGF-R2^+^ EPCs fraction yielded 135.3 ± 68.1 ng/μl RNA from diabetic mice and 88.0 ± 53.3 ng/μl RNA from non-diabetic mice (p = 0.05). The integrity of RNA, expressed as RNA quality indicator (RQI) was ≥7 in all samples, indicating intact RNA (see BioRad tech note 5761 Rev B; http://www.biorad.com).

### Gene expression changes

Of the 35 genes studied, HSPD1, SDF-1 and SELE showed significant changes between non-diabetic and diabetic Lin^-^/VEGF-R2^+^ EPCs. While SDF-1 was down regulated about 0.3-fold, SELE was up regulated 2.4-fold in diabetic compared to non-diabetic Lin^-^/VEGF-R2^+^ EPCs. No significant changes in SDF-1 and SELE expression were found between non-diabetic and diabetic Lin^+^ cells, whereas HSPD1 showed a significant change and a similar fold-change in the diabetic Lin^+^ cells compared to the non-diabetic Lin^+^ cells. Therefore, we considered that the differential expression of SDF-1 and SELE genes was specific for Lin^-^/VEGF-R2^+^ EPCs. There was a small non-significant increase in the expression of CXCR4 between non-diabetic and diabetic Lin^-^/VEGF-R2^+^ EPCs. HIF1A and Tie2 were significantly differentially expressed between diabetic Lin^+^ cells and non-diabetic Lin^+^ cells only. Unfortunately, no analysis could be performed for 6 genes including CAV1, eNOS, CLDN5, NANOG, OCLN and BDNF due to CT values ≥35. It is accepted that CT values of 35 or higher represent detection of single molecules in the sample, these readings are therefore considered noise, hence are not reliable expression values and should not be analysed [34-36]. A detailed overview of the results of the gene-expression analysis is shown in Table 1.

**Table 1:** Overview of the RT-PCR analysis

	**Diabetic lin**^**+ **^**vs. Non-diabetic lin**^**+**^			**Diabetic EPC vs. Non-diabetic EPC**		
**Gene**	**Fold-change**	**95% CI of fold-change**	**p-value**	**Fold-change**	**95% CI of fold-change**	**p-value**
**AKT**	1.04	(0.84, 1.24)	0.7622	0.34	(0.00, 1.16)	0.9111
**BDNF**	-	-	-	-	-	-
**CASP9**	0.77	(0.54, 1.00)	0.1172	0.79	(0.55, 1.03)	0.1151
**CAV1**	-	-	-	-	-	-
**CDH5**	0.84	(0.62, 1.07)	0.1977	0.81	(0.30, 1.32)	0.8824
**CLDN5**	-	-	-	-	-	-
**CXCR4**	0.73	(0.50, 0.96)	0.0800	0.80	(0.54, 1.06)	0.1993
**eNOS**	-	-	-	-	-	-
**EPO**	1.06	(0.62, 1.51)	0.8700	1.16	(0.68, 1.64)	0.5543
**EPO-R**	1.21	(0.80, 1.61)	0.1860	0.90	(0.68, 1.13)	0.5005
**FGF1**	0.73	(0.08, 1.38)	0.3329	0.76	(0.17, 1.35)	0.2288
**FN1**	1.24	(0.72, 1.77)	0.5181	1.62	(0.94, 2.29)	0.0628
**GATA2**	0.73	(0.47, 0.99)	0.1182	0.62	(0.32, 0.93)	0.0534
**HIF1A**	0.52	(0.35, 0.69)	0.0019	0.78	(0.35, 1.20)	0.3114
**HOXA9**	1.29	(0.90, 1.68)	0.0890	0.65	(0.00, 1.72)	0.4628
**HSPD1**	0.51	(0.35, 0.68)	0.0005	0.59	(0.32, 0.86)	0.0371
**ICAM1**	0.95	(0.67, 1.22)	0.6373	0.70	(0.39, 1.01)	0.0997
**IGF1**	0.89	(0.18, 1.60)	0.6833	1.02	(0.14, 1.90)	0.9718
**IL11**	1.48	(0.74, 2.23)	0.1445	1.70	(0.64, 2.77)	0.1572
**IL6**	1.04	(0.84, 1.25)	0.7680	1.33	(0.90, 1.75)	0.1526
**MMP2**	0.88	(0.38, 1.38)	0.5561	0.88	(0.35, 1.41)	0.5012
**MMP9**	0.78	(0.40, 1.17)	0.4096	1.05	(0.76, 1.34)	0.6447
**NANOG**	-	-	-	-	-	-
**OCLN**	-	-	-	-	-	-
**P53**	0.92	(0.79, 1.05)	0.2973	1.05	(0.89, 1.22)	0.6169
**PIK3R1**	0.80	(0.56, 1.04)	0.2263	0.81	(0.60, 1.02)	0.1029
**PKC**	1.04	(0.77, 1.30)	0.7644	1.08	(0.82, 1.33)	0.6184
**PTPN11**	0.94	(0.77, 1.11)	0.5361	1.14	(0.96, 1.32)	0.1280
**SDF-1**	0.48	(0.16, 0.80)	0.0891	0.32	(0.04, 0.60)	0.0149
**SELE**	1.92	(0.24, 3.60)	0.1913	2.41	(1.42, 3.39)	0.0005
**Tie2**	2.28	(0.89, 3.68)	0.0059	1.22	(0.69, 1.75)	0.5184
**VCAM1**	1.22	(0.85, 1.60)	0.2085	0.62	(0.25, 0.98)	0.0687
**VEGFA**	1.08	(0.00, 3.69)	0.9400	1.12	(0.83, 1.41)	0.4433
**VEGFR1**	1.03	(0.70, 1.35)	0.8699	0.59	(0.25, 0.93)	0.0729
**VEGFR2**	0.56	(0.26, 0.86)	0.0721	0.55	(0.14, 0.96)	0.2806

The table indicates the changes in diabetic vs non-diabetic. Down regulated genes have a fold-change < 1 and up regulated genes have a fold-change > 1. Only SDF-1 and SELE show significant changes in the Lin^-^/VEGF-R2^+^ EPCs while showing no significant changes in the Lin^+^ group. HIF1A shows significant change in the Lin^+^ group but not in the Lin^-^/VEGF-R2^+^ EPCs group.

### Protein level changes

**Figure 2:**
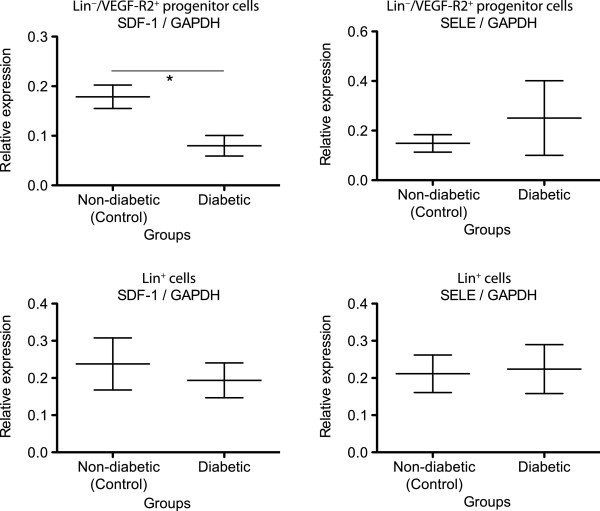
**Relative expression of SDF-1 and SELE in relation to GAPDH expression (reference gene) in Lin**^**-**^**/VEGF-R2**^**+ **^**EPCs and Lin**^**+ **^**cells.** The central bar indicates the mean, whiskers indicates ± 1 SD interval. Asterisk (*) denotes statistical significance (p < 0.05).

**Figure 3:**
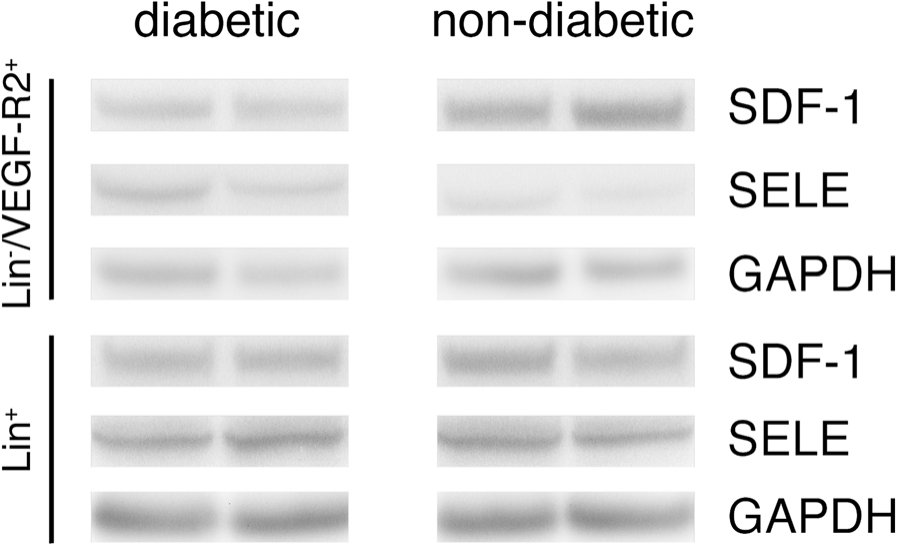
**Western blot membrane imaging.** Protein expression of SDF-1 and SELE in Lin^-^/VEGF-R2^+^ EPCs and Lin^+^ cells isolated from the BM of non-diabetic and diabetic mice. Specific SDF-1 and SELE signal was detected by probing blots with anti-SDF-1 and anti-SELE antibodies. Two representative samples of a total of eight biological replicates per group are shown. GAPDH was used as loading control. Densitometry analysis was processed to evaluate the expression of SDF-1 and SELE. While Lin^+^ cells do not show obvious changes, Lin^-^/VEGF-R2^+^ EPCs show changes regarding SDF-1 and SELE expression, though only changes observed in SDF-1 are statistically significant.

As only SDF-1 and SELE showed specific changes in Lin^-^/VEGF-R2^+^ EPCs in diabetic animals compared with non-diabetics, validation of differential expression at the protein level was performed only for these two genes. For the two genes studied, Western blot showed that only SDF-1 was significantly down regulated in diabetic BM Lin^-^/VEGF-R2^+^ EPCs, while no significant change was found for SELE in Western blot analysis (see Figures 2 and 3, Table 2).

**Table 2:** Overview of the Western blot analysis

	**Lin**^**+**^		**Lin**^**-**^**/VEGF-R2**^**+**^	
	**Diabetic vs Non-diabetic**		**Diabetic vs Non-diabetic**	
	**Fold-change**	**p-value**	**Fold-change**	**p-value**
**SELE**	1.06	0.676	1.69	0.2136
**SDF-1**	0.86	0.1645	0.45	0.0114

Only SDF-1 shows significant changes on the protein level while the up-regulation of SELE is not significant.

## Discussion

The present study analyzed differential gene expression in freshly isolated Lin^-^/VEGF-R2^+^ EPCs obtained from murine BM in the early stages of diabetes (18 weeks). A total of 35 genes that were previously reported to be involved in EPC mobilization and EPC function [15,37-57] were tested to see whether the reported diabetes-related changes observed mainly in EPCs from PB of diabetic humans could also be found in BM-derived progenitor cells from mice with early diabetes. There were three main findings. Firstly, the number of Lin^-^/VEGF-R2^+^ EPCs/g body weight within the BM was significantly higher in diabetic than non-diabetic animals. Secondly, SDF-1 and SELE were significantly differentially expressed in diabetic Lin^-^/VEGF-R2^+^ EPCs but not in diabetic BM Lin^+^ cells, indicating that the differential expression of SDF-1 and SELE are specific for BM Lin^-^/VEGF-R2^+^ EPCs in diabetic mice. The changes observed at the mRNA level were confirmed by Western blot analysis only for SDF-1. Thirdly, 6 genes including CAV1, eNOS, CLDN5, NANOG, OCLN and BDNF showed such low levels of expression that no comparison could be made. These results demonstrate differential expression of one gene (SDF-1) of BM derived progenitor cells in diabetic mice that may contribute to their dysfunctional mobilization from the BM to the PB and that Lin^-^/VEGF-R2^+^ EPCs may be very early progenitor cells.

The observation of higher numbers of Lin^-^/VEGF-R2^+^ EPCs/g body weight found in diabetic mice compared with non-diabetic animals is consistent with previous findings in a streptozotocin-induced diabetic mouse model [28]. This may indicate that in early stages of diabetes progenitors are "trapped" inside the BM. The reduced expression of SDF-1, one of the key factors involved in EPC mobilization may partially contribute to this finding. Based on the current results, it seems in early diabetes mainly impacts mobilization rather than cell genuine progenitor cell function, which would tally reports that reduction of EPCs in the PB in patients with diabetes is not necessarily coupled to an impaired function [58].

The ability of EPCs to produce SDF-1 has been shown in EPCs isolated from the BM [59] as well as in those isolated from the PB [60]. As EPCs express both SDF-1 and its receptor (CXCR4) [61], an autocrine/paracrine regulation loop for SDF-1 [62] within EPCs has been proposed. The functional interaction between SDF-1 and CXCR4 in EPCs within the BM is still unclear. However, recent studies indicate that the interaction between SDF-1 ligand and its CXCR4 receptor appears to play important roles in both mobilization of EPCs from the BM to the PB and EPC maturation [62,63]. Our finding of down regulation of SDF-1 in diabetic BM Lin^-^/VEGF-R2^+^ EPCs indicates that reduced expression of SDF-1 may contribute to the impaired mobilization of Lin^-^/VEGF-R2^+^ EPCs observed in diabetic mice. A recent study proposed that high glucose leads to reduced expression of HIF1α which in turn results in a lower level of SDF-1 expression [64]. Others have reported that advanced glycation end products impair SDF-1 production in endothelial progenitor cells in a dose dependent manner [65].

Consistent with these findings of impaired mobilisation of Lin^-^/VEGF-R2^+^ EPCs and SDF-1 downregulation are recent reports that SDF-1 is crucial in mobilizing progenitor cells [66] and that direct application of SDF-1 to rats with myocardial infarction reduced the infarction size, probably by stimulating migration of progenitor cells to the heart thereby altering postinfarction vascular remodeling [67]. These recent findings together with the results of the current study emphasize the importance of an early SDF-1 downregulation on cell mobilisation. The interactions between HIF1α expression, regulation of SDF-1 expression and mobilization of BM-EPCs in diabetic vasculopathies warrant further research.

Expression of SELE (CD62E) in circulating EPCs has been described [68,69]. Several recent studies suggest that SELE expression is a sign of EC or EPC activation [70-72] because unstimulated BM-derived EPCs do not express SELE [73]. The up-regulation of SELE in Lin^-^/VEGF-R2^+^ EPCs in diabetic BM may be attributed to increased production of inflammatory cytokines such as interleukin 1 and tumor necrosis factors caused by the diabetic condition [74].

Many studies have linked the EPC function to eNOS, which is considered one of the cardinal enzymes involved in mobilization of EPCs from the BM to the PB as well as maintaining the normal function of EPCs in physiological conditions [41,42,75-77]. In the present study we found that eNOS expression in BM Lin^-^/VEGF-R2^+^ EPCs was very low. This is in accordance with a number of observations showing that eNOS expression is absent in immature EPCs but its level is increased when EPCs become more mature [43,44,77]. The low level of eNOS as well as CAV1, CLDN5, NANOG, OCLN and BDNF expression in BM Lin^-^/VEGF-R2^+^ EPCs may indicate that the Lin^-^/VEGF-R2^+^ population consists mainly of immature or very early progenitor cells.

Mobilization of progenitor cells from the BM is complex and is based on the interplay of different factors. Results from testing 35 genes for differential expression can explain only partially observed effects of diabetes on BM progenitor cells, which can be considered as a limitation of this study. Further studies may choose broader approaches. Another limitation is that gene expression was tested only at one time point and hence no information on the timing of the SDF-1 expression changes can be monitored or whether other genes may be differentially expressed in the course of diabetes impacting mobilization and/or function.

## Conclusions

Overall, the present study indicates that a short period of diabetes is sufficient to trap a significant number of Lin^-^/VEGF-R2^+^ EPCs in the BM and to induce reduced expression of SDF-1, which could be one of the predominant reasons to account for the dysfunctional mobilization of Lin^-^/VEGF-R2^+^ EPCs from the BM to PB. Future research on regulation of SDF-1 expression in BM-EPCs may lead to novel therapeutic strategies for treatment of early diabetic vasculopathies.

## Abbreviations

BM: Bone marrow; BSA: Bovine serum albumin; DiI-Ac-LDL: 1, 1\'-dioctadecyl – 3, 3, 3\', 3\'-tetramethyl-indocarbocyanine perchlorate acetylated low density lipoprotein; ECs: Endothelial cells; EPCs: Endothelial progenitor cells; GAPDH: Glyceraldehyde-3-phosphate dehydrogenase; Lin^-^/VEGF-R2^+^ EPCs: Lin^-^/VEGF-R2^+^ endothelial progenitor cells; Lin: Hematopoietic lineage; PB: Peripheral blood; PVDF: Polyvinyl difluoride membrane; SD: Standard deviation; SDF-1: Stromal derived factor-1; VEGF-R2: Vascular endothelial growth factor-receptor 2.

## Competing interests

The authors confirm that there are no competing interests.

## Authors’ contributions

DB participated in the design of the study, participated in animal work, BM collection, cells, RNA and protein isolation, RT-PCR, WB, statistical analysis and drafted the manuscript. LZ participated in RT-PCR and WB. WS and MCG conceived of the study, and participated in its design and helped to draft the manuscript. MRI conceived of the study, participated in its design, participated in animal work, BM collection, cells isolation, statistical analysis and helped to draft the manuscript. All authors read and approved the final manuscript.

## Authors’ information

Save Sight Institute, Sydney Hospital and Sydney Eye Hospital, Central Clinical School, The University of Sydney, Sydney, NSW 2000 Australia.

## Supplementary Material

Additional file 1: Table S1 Overview of the 35 genes tested, allocated to either EPC mobilization and/or EPC function, the primers used for RT-PCR. Click here for file
