# Supplementary material for: Differential gene expression in Lin-/VEGF-R2+ bone marrow-derived endothelial progenitor cells isolated from diabetic mice
Source: Cardiovasc Diabetol. 2014 Feb 12;13:42. doi: 10.1186/1475-2840-13-42 (PMC3926942; doi:10.1186/1475-2840-13-42)
Supplement: Additional file 1: Table S1 — Overview of the 35 genes tested, allocated to either EPC mobilization and/or EPC function, the primers used for RT-PCR. [file 1475-2840-13-42-S1.doc]

**Supplementary Material**

**Table S1: Overview of the 35 genes tested, allocated to either EPC mobilization and/or EPC function, the primers used for RT-PCR.**

| Functional Group | Gene Symbol | Gene name | Forward / Reverse Primer |
| --- | --- | --- | --- |
| mobilization | MMP2 | matrix metalloproteinase 2 | CAAGTTCCCCGGCGATGTC  TTCTGGTCAAGGTCACCTGTC |
|  | MMP9 | matrix metalloproteinase 9 | CTGGACAGCCAGACACTAAAG  CTCGCGGCAAGTCTTCAGAG |
|  | CXCR4 | chemokine receptor type 4 | GAAGTGGGGTCTGGAGACTAT  TTGCCGACTATGCCAGTCAAG |
|  | CAV1 | caveolin 1 | TGTATGACGCGCACACCAA  TGGTTCTGCAATCACATCTTCAA |
|  | SDF-1 | stromal cell-derived factor-1 | TGCATCAGTGACGGTAAACCA  TTCTTCAGCCGTGCAACAATC |
|  | eNOS | endothelial nitric oxide synthase | GGTCCTGTGCATGGATGAG  GTTGTACGGGCCTGACATTT |
|  | ICAM1 | intercellular adhesion molecule 1 | GTGATGCTCAGGTATCCATCCA  CACAGTTCTCAAAGCACAGCG |
|  | VEGFR1/Flt1 | vascular endothelial growth factor receptor 1 | CCACCTCTCTATCCGCTGG  ACCAATGTGCTAACCGTCTTATT |
|  | VEGFR2/Flk1 | vascular endothelial growth factor receptor 2 | TTTGGCAAATACAACCCTTCAGA  GCAGAAGATACTGTCACCACC |
|  | HIF1A | hypoxia-inducible factor-1 | ACCTTCATCGGAAACTCCAAAG  CTGTTAGGCTGGGAAAAGTTAGG |
|  | VCAM 1 | vascular cell adhesion protein 1 | AGTTGGGGATTCGGTTGTTCT  CCCCTCATTCCTTACCACCC |
|  | IL6 | interleukin 6 | GCCACCGTTACCCTGATTTG  CCAGAGTACACCCAGTGAATGG |
|  | EPOR | erythropoietin receptor | CAACAGCGGACACATCGAGTT  TGCGGTGATAGCGAGGAGA |
|  | EPO | erythropoietin | AGGAATTGATGTCGCCTCCA  AGCTTGCAGAAAGTATCCACTGTG |
|  | PTPN11 | protein tyrosine phosphatase SHP2 | ATGACATCGCGGAGATGGTTT  GGGTTACTCTTACTGGGCCTT |
|  | VEGFA | vascular endothelial growth factor A | GCACATAGAGAGAATGAGCTTCC  CTCCGCTCTGAACAAGGCT |
|  | SELE | E-selectin | ATGCCTCGCGCTTTCTCTC  GTAGTCCCGCTGACAGTATGC |
|  | AKT | protein kinase B | GCACCTTTATTGGCTACAAGGA  GGGGACTCTCGCTGATCCA |
|  | BDNF | brain-derived neurotrophic factor | TCATACTTCGGTTGCATGAAGG  GTCCGTGGACGTTTACTTCTTT |
| function | CASP9 | caspase 9 | TCCTGGTACATCGAGACCTTG  AAGTCCCTTTCGCAGAAACAG |
|  | HSPD1 | heat shock protein 1 | CACAGTCCTTCGCCAGATGAG  CTACACCTTGAAGCATTAAGGCT |
|  | PIK3R1 | phosphatidylinositol 3-kinase regulatory subunit alpha | CCCACTACTGTAGCCAACAAC  CGTACCAAAAAGGTCCCATCA |
|  | GATA 2 | GATA binding protein 2 | CACCCCGCCGTATTGAATG  CCTGCGAGTCGAGATGGTTG |
|  | eNOS | endothelial nitric oxide synthase | GGTCCTGTGCATGGATGAG  GTTGTACGGGCCTGACATTT |
|  | VEGFR2Flk1 | vascular endothelial growth factor receptor 2 | TTTGGCAAATACAACCCTTCAGA  GCAGAAGATACTGTCACCACC |
|  | CLDN5 | claudin 5 | GCAAGGTGTATGAATCTGTGCT  GTCAAGGTAACAAAGAGTGCCA |
|  | CDH5 | vascular endothelial cadherin | AGGACAGCAACTTCACCCTCA  AACTGCCCATACTTGACCGTG |
|  | IL6 | interleukin 6 | GCCACCGTTACCCTGATTTG  CCAGAGTACACCCAGTGAATGG |
|  | Tie-2 | angiopoietin receptor 2 | CGGCCAGGTACATAGGAGGAA  TCACATCTCCGAACAATCAGC |
|  | FN1 | fibronectin | ATGTGGACCCCTCCTGATAGT  GCCCAGTGATTTCAGCAAAGG |
|  | P53 | protein 53 | GCGTAAACGCTTCGAGATGTT  TTTTTATGGCGGGAAGTAGACTG |
|  | PKC | protein kinase C | GTTTACCCGGCCAACGACT  GGGCGATGAATTTGTGGTCTT |
|  | NANOG | homeobox protein NANOG | TCTTCCTGGTCCCCACAGTTT  GCAAGAATAGTTCTCGGGATGAA |
|  | FGF1 | heparin-binding growth factor 1 | CAGCTCAGTGCGGAAAGTG  TGTCTGCGAGCCGTATAAAAG |
|  | IGF1 | insulin-like growth factor 1 | CTGGACCAGAGACCCTTTGC  GGACGGGGACTTCTGAGTCTT |
|  | AKT | protein Kinase B | GCACCTTTATTGGCTACAAGGA  GGGGACTCTCGCTGATCCA |
|  | HOXA9 | homeobox protein Hox-A9 | GCCACACGAACCAAGAGGAC  CGGGTGCGTACATAGAGCATAA |
|  | BDNF | brain-derived neurotrophic factor | TCATACTTCGGTTGCATGAAGG  GTCCGTGGACGTTTACTTCTTT |
|  | IL11 | interleukin 11 | GGCCAGATAGAGTCGTTGCC  GGGATCGGGTTAGGAGAACAG |
|  | OCLN | occludin | TTGAAAGTCCACCTCCTTACAGA  CCGGATAAAAAGAGTACGCTGG |
